# Serum level of S100A8/A9 as a biomarker for establishing the diagnosis and severity of community-acquired pneumonia in children

**DOI:** 10.3389/fcimb.2023.1139556

**Published:** 2023-04-27

**Authors:** Si Xie, Jun Wang, Wenbin Tuo, Shihao Zhuang, Qinzhen Cai, Cong Yao, Feng Han, Hongmin Zhu, Yun Xiang, Chunhui Yuan

**Affiliations:** ^1^ Department of Laboratory Medicine, Wuhan Children’s Hospital (Wuhan Maternal and Child Healthcare Hospital), Tongji Medical College, Huazhong University of Science and Technology, Wuhan, China; ^2^ Fujian Maternity and Child Health Hospital, Affiliated Hospital of Fujian Medical University, Fuzhou, China; ^3^ Health Care Department, Wuhan Children’s Hospital (Wuhan Maternal and Child Healthcare Hospital), Tongji Medical College, Huazhong University of Science and Technology, Wuhan, China; ^4^ Department of Pediatric Respiratory Medicine, Wuhan Children’s Hospital (Wuhan Maternal and Child Healthcare Hospital), Tongji Medical College, Huazhong University of Science and Technology, Wuhan, China; ^5^ Department of Neurology, Wuhan Children’s Hospital (Wuhan Maternal and Child Healthcare Hospital), Tongji Medical College, Huazhong University of Science and Technology, Wuhan, China

**Keywords:** S100A8/A9, severity, biomarker, community-acquired pneumonia, children

## Abstract

**Background:**

S100A8/A9, which is a member of S100 proteins, may be involved in the pathophysiology of Community-acquired pneumonia (CAP) that seriously threatens children’s health. However, circulating markers to assess the severity of pneumonia in children are yet to be explored. Therefore, we aimed to investigate the diagnostic performance of serum S100A8/A9 level in determining the severity of CAP in children.

**Methods:**

In this prospective and observational study, we recruited 195 in-hospital children diagnosed with CAP. In comparison, 63 healthy children (HC) and 58 children with non-infectious pneumonia (pneumonitis) were included as control groups. Demographic and clinical data were collected. Serum S100A8/A9 levels, serum pro-calcitonin concentrations, and blood leucocyte counts were quantified.

**Results:**

The serum S100A8/A9 levels in patients with CAP was 1.59 ± 1.32 ng/mL, which was approximately five and two times higher than those in healthy controls and those in children with pneumonitis, respectively. Serum S100A8/A9 was elevated parallelly with the clinical pulmonary infection score. The sensitivity, specificity, and Youden’s index of S100A8/A9 ≥1.25 ng/mL for predicting the severity of CAP in children was optimal. The area under the receiver operating characteristic curve of S100A8/A9 was the highest among the indices used to evaluate severity.

**Conclusions:**

S100A8/A9 may serve as a biomarker for predicting the severity of the condition in children with CAP and establishing treatment grading.

## 1 Introduction

Community-acquired pneumonia (CAP) is a major cause of morbidity and mortality worldwide, particularly among children aged younger than 5 years old (Liu et al., 2015). Therefore, many studies have focused on the diagnosis or therapeutics of CAP. In the research about the diagnosis and treatment of CAP, it has been found that severe CAP causes serious complications, while its mortality rate is more than 10%, which is significantly higher than that of mild CAP (Rudan et al., 2008, Rudan et al., 2010; Koh et al., 2017). Accordingly, assessing the presence and severity of CAP is essential for clinicians to make more accurate treatment choices and allocate resources more effectively (Dagan et al., 2011). Currently, several clinical and analytical scales have been established to evaluate the severity and prognosis of CAP, such as the Pneumonia Severity Index and CURB-65 scores only for adults (Lim et al., 2003). However, data on predicting the severity of children with CAP are limited, and no common respiratory clinical scoring models exist for the pediatric patient population (Chiang et al., 2007).

The current guidelines are based only on assessing clinical signs and symptoms; therefore, clinicians may underestimate/overestimate the severity of the condition in children (Bradley et al., 2011; Harris et al., 2011; Organization, 2013). Since CAP is a lung injury caused by an out-of-hospital (community) pathogen infection, its severity depends on the pathogenicity, load, virulence of the microorganism, and the immune response to the infection (Bermejo-Martin et al., 2017). Based on this concept, several approaches have been developed to classify the severity of CAP in children, such as radiationless lung ultrasound analysis, artificial intelligence-based analysis of computed tomography and chest X-ray images, evaluation of the viral load using polymerase chain reaction, and the identification of specific clinical signs (Zieleskiewicz et al., 2020). However, the empirical level of practice required for these methods or their high cost are barriers to widespread clinical use (Florin et al., 2021). Therefore, there is a growing interest in identifying easily accessible circulating host biomarkers alone or in combination to improve the risk/severity stratification.

Available laboratory indicators of the severity of inflammation include C-reactive protein (CRP), procalcitonin (PCT), interleukin 6 (IL-6), serum amyloid A (SAA), ferritin (FER), neutrophil CD64, and heparin-binding protein (HBP), among others, whose values are indicative of the type or severity of infection. These indicators are partly secreted by the intrinsic immune cells, and S100A8/A9 is one of the similar indicators. S100A8/A9, also called myeloid-related protein 8/14, is the heterodimer formed by S100A8 and S100A9, which belongs to the Ca2^+^ binding S100 protein family (Pruenster et al., 2016). When pathogens invade, innate immunity is activated; subsequently, neutrophils and phagocytes secrete S100A8/A9 (De Filippo et al., 2014). As a cytokine that activates T and B cells, S100A8/A9 initiates acquired immunity and induces an inflammatory cascade response (Wang et al., 2018). Meanwhile, S100A8/A9 is the most abundant damage-associated molecular pattern (DAMP) molecule that triggers an inflammatory cascade response, leading to cell death and tissue damage in many inflammatory diseases (Chen et al., 2015). Based on these mechanisms, S100A8/A9 expression is elevated in patients with infectious diseases, including periodontitis (Karna et al., 2019), cervical inflammation (Kunimi et al., 2006), peritonitis (Schwarz et al., 2007), and purulent infections (Van Zoelen et al., 2009). Furthermore, recent studies have demonstrated that serum S100A8/A9 levels correlate with the severity and prognosis of coronavirus disease 2019 (Chen et al., 2020; Ren et al., 2021). Nevertheless, data on the role of this biomarker in diagnosis, severity, and prognosis in childhood CAP are limited.

Therefore, this study aimed to determine the levels of serum S100A8/A9 in children with CAP and to analyze its relationship with the baseline clinical characteristics and laboratory data for the diagnosis and severity evaluation of CAP.

## 2 Materials and methods

### 2.1 Participants

**Figure 1:**
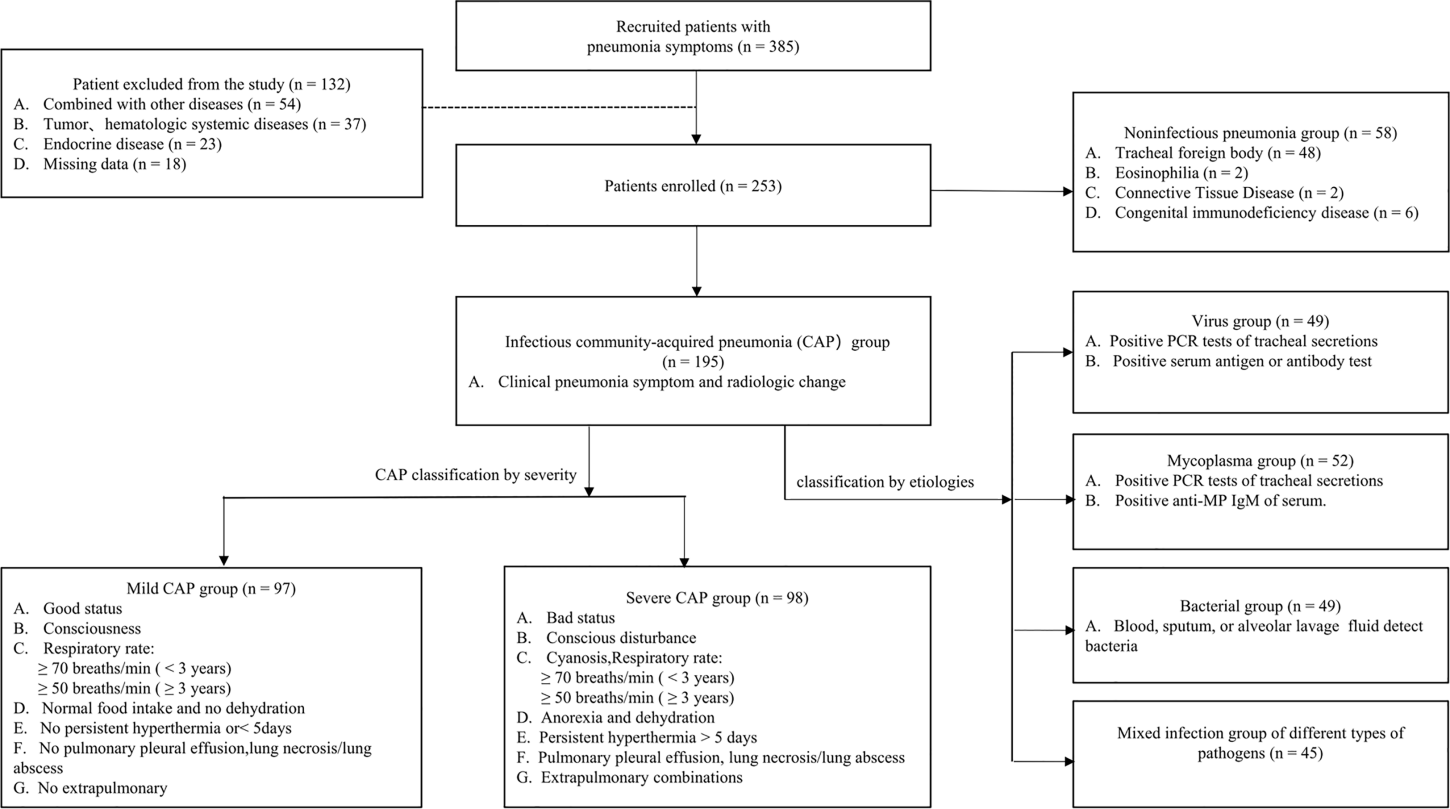
Flow diagram of recruitment and follow-up research in this cohort study.

This was a prospective, open-label, non-blinded observational study on 195 hospitalized patients with CAP who were admitted to the Wuhan Children’s Hospital, Tongji Medical College Huazhong University of Science and Technology, from January 1, 2022, to July 30, 2022. Meanwhile, 58 patients with non-infection pneumonia (pneumonitis) and 63 healthy individuals who visited the physical examination center and had no respiratory tract infection within the preceding 3 months were recruited as control groups. Additionally, demographic and clinical data were collected from Electronic Medical Record System on admission. Patients were included if they fulfilled the following criteria: age between 1 month to 18 years, presence of fever and respiratory symptoms, and having at least one abnormality in the physical examination or chest radiographs according to the guidelines for CAP in children (Commission, 2019). We also distinguished the severity of CAP according to this guideline and combined it with the clinician’s diagnosis. The exclusion criteria were as follows: congenital heart disease, tumor, hematologic systemic diseases, endocrine disease, neuromuscular disease, immunodeficiency, or a combination of other diseases, including any chronic disease that may promote the development of severe pneumonia (cystic fibrosis, asthma, bronchopulmonary dysplasia, tuberculosis, renal disease, and inflammatory bowel disease, among others). Moreover, patients were excluded if their parents or guardians did not provide proxy consent or if data were missing. The specific recruitment process and grouping basis are shown in **Figure 1**.

Written informed consent was obtained from all parents. Ethics approval was reviewed and provided by the Medical Ethical Committee of the Wuhan Children’s Hospital, Huazhong University of Science and Technology (2022R048-E01).

### 2.2 Laboratory measurements

Complete blood count, PCT, and hypersensitive CRP (hs-CRP) were collected from the children recruited upon admission. The measurements were performed in our central and biochemistry laboratory. A complete blood count was performed using an automated analyzer (Mindray 7500CS, Shenzhen, China). In addition, serum hs-CRP and PCT were measured using nephelometry with a commercially available assay (Siemens ADVIA 2120i, Erlangen, Germany). The normal reference ranges for hs-CRP and PCT were 0–3 mg/L and < 0.05 ng/mL, respectively.

Enzyme-linked immunosorbent assay (HYCEZMBIO, Cat# HY10208) was used to measure the S100A8/A9 levels. According to the manufacturer’s instructions, venous blood was centrifugated for quantifying serum S100A8/A9 concentrations, and serum was immediately stored at -80°C until assayed. A 50 μL serum sample was needed to measure S100A8/A9 concentration. Assays were conducted in triplicate on each standard or sample according to a standard curve generated by diluting recombinant S100A8/A9 protein. There was a detection limit of 0.01 ng/mL; the coefficient of variation within and between assays was 4.5%–10% and 5%–15%, respectively, for concentrations ranging from 0.125 to 4 ng/mL.

### 2.3 Statistical analysis

Statistical analyses were performed using SPSS version 25.0 (SPSS Inc., Chicago, IL, USA) and MedCalc version 19.1.3 (Mariakerke, Belgium). Continuous variables are expressed as means ± standard deviations and median (inter-quartile range) for normally distributed and skewed data, respectively. Categorical variables are presented as counts (percentages). Chi-square tests were used to compare qualitative variables. Furthermore, Spearman correlation analysis was used to analyze correlations. The Mann–Whitney U-test and chi-square test were used for two-group comparisons, and the Kruskal–Wallis test was used for multi-group comparisons. The performance of biomarkers in predicting pneumonia severity was assessed using the receiver operating characteristic. Subsequently, the area under the curve (AUC) was calculated with 95% confidence intervals. Cut-off values of the biomarkers were determined based on sensitivity and specificity. Logistic regression was used to assess the ability of demographic characteristics, clinical findings, and laboratory values to efficiently predict the risk of severity. Two-sided *P* < 0.05 showed statistical significance.

## 3 Results

### 3.1 Demographic characteristics and clinical information

Overall, 195 patients with CAP, 58 with pneumonitis, and 63 healthy individuals were included. The demographic and clinical characteristics were analyzed. As presented in **Table 1**, no significant difference was observed in gender and age among the groups (*P* > 0.05). Patients with CAP were categorized into four groups based on the different types of pathogens, which are listed along with 58 patients with pneumonitis in **Table 2**. The analysis revealed higher hs-CRP and PCT levels in patients with CAP infected with bacteria than in those in the groups with other types of CAP but lower levels than those in the group with pneumonitis.

**Table 1:** General information of CAP group and control groups.

	CAP group (n = 195)	Healthy group (n = 63)	Pneumonitis group (n = 58)	*P-*value
**Ages(months)**	39.23 ± 30.23	42.81 ± 20.47	39.00 ± 37.93	0.694
**Sex, n (%)**				0.264
**male**	121 (62.05)	32 (50.79)	36 (62.07)	
**female**	74 (37.95)	31 (49.21)	22 (37.93)	

Data comparison used χ^2^ Chi-squared test and One-way ANOVA test. P < 0.05 was considered statistically significant.

**Table 2:** Differences between groups of pneumonia due to different etiologies.

	Bacterial group (n = 49)	Virus group (n = 49)	Mycoplasma group (n = 52)	MIX group (n = 45)	Pneumonitis group (n = 58)	*P-*value
**Mild, n (%)**	23 (47.94)	24 (48.98)	26 (50.00)	23 (51.11)	**/^a^**	0.991
**Severe, n (%)**	25 (52.06)	25 (51.02)	26 (50.00)	22 (48.89)	**/^a^**	0.991
**hs-CRP (mg/L)**	19.8 (6.5, 32.8)	5.63 (1.01, 13.99)	10.32 (2.9, 26.7)	7.6 (1.21, 23.1)	34.2 (10.2, 66.5)	<0.001
**PCT (ng/mL)**	0.17 (0.08, 0.57)	0.09 (0.06, 0.14)	0.11 (0.05, 0.3)	0.14 (0.08, 0.52)	0.2 (0.06, 1.76)	0.004

Mild, Mild community-acquired pneumonia group; Severe, Severe community- acquired pneumonia group; MIX, Mixed infections with two or more different types of pathogens; hs-CRP, hypersensitive C-reactive protein; PCT, pro-calcitonin; /^a^, The severity of Pneumonitis not classified. Data comparison used χ^2^ Chi-squared test and Kruskal-Wallis test. P < 0.05 was considered statistically significant.

### 3.2 Serum S100A8/A9 levels in control individuals and children with CAP

**Figure 2:**
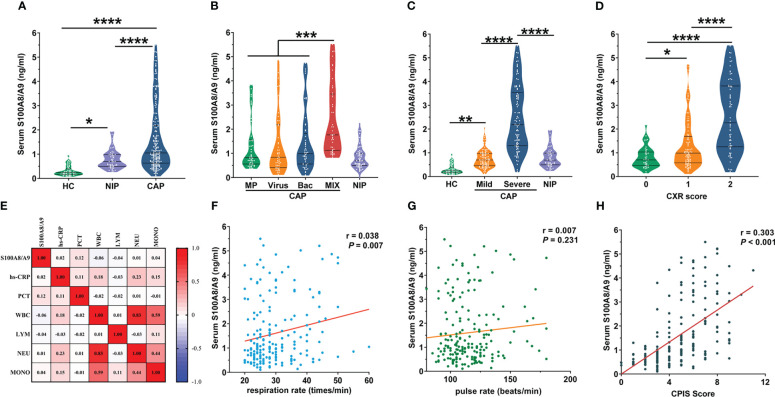
The levels of serum S100A8/A9 between groups and its correlation with other indices. **(A)** Serum S100A8/A9 in CAP patients and control subjects. **(B)** Due to the different types of pathogens causing CAP, based on the corresponding pathogenic test results, we divided the 195 children with CAP into four groups: bacterial group (n = 49), viral group (n = 49), mycoplasma group (n = 52) with a single infection type, and mixed infection group (n = 45) with more than two infection types. The graph shows serum S100A8/A9 in different infected etiologies of CAP patients and control groups. **(C)** The 195 recruited pediatric patients with CAP divided into mild group (n = 97) and severe group (n = 98) based on the criteria of the guideline of CAP in children (Commission, 2019). The graph shows serum S100A8/A9 in different severity of CAP patients and control subjects. **(D)** Correlation of serum S100A8/A9 with common laboratory indicators of the severity of inflammation. **(E)** Serum S100A8/A9 in different Chest radiography (CXR) scores in CAP children. **(F, G)** Correlation of serum S100A8/A9 with respiration rate and pulse rate. **(H)** Correlation of serum S100A8/A9 with CPIS Scores. Serum S100A8/A9 was detected using ELISA. All data were expressed as mean ± SEM. Correlations were analyzed using the Spearman correlation analysis. *P < 0.05, **P < 0.01, ***P < 0.001, ****P < 0.0001.

Serum S100A8/A9 levels were measured and compared between controls and patients with CAP. The S100A8/A9 levels increased in children with CAP and patients with non-infection pneumonia. However, the S100A8/A9 levels in children with CAP showed more significant elevation than in the patients with non-infection pneumonia (**Figure 2A**). The serum S100A8/A9 levels were further compared among the groups infected with different pathogen types in patients with CAP. The results indicated that among the patients with the same severity of CAP, serum S100A8/A9 levels did not differ between those with CAP with single infections and those infected with different types of pathogens (**Figure 2B**). However, the plasma S100A8/A9 levels in patients with CAP who were infected with two or more types of pathogens were increasing compared with that in those who were infected with only one type of pathogen. Meanwhile, children with pneumonia infected with bacteria had higher serum S100A8/A9 levels than those with non-infectious pneumonia. S100A8/A9 expression was significantly higher in the group of children who had severe CAP than that in the control groups; however, S100A8/A9 level in the group with mild CAP did not differ from that in the non-infection group (**Figure 2C**).

### 3.3 Associations of serum S100A8/A9 with the severity of CAP in children

We categorized children with CAP into mild and severe groups according to the disease severity. Since the expression of S100A8/A9 was significantly higher in the severe group than that in the mild group (**Figure 2C**), additional factors were measured to identify other differences between the mild and severe groups and the correlation among these differences with S100A8/A9.

#### 3.3.1 Clinical and laboratory characteristics of children with mild and severe CAP

The clinical characteristics and laboratory findings in both groups are presented in **Table 3**. The paroxysm duration before admission, duration of fever before admission, and length of stay in the severe CAP group were significantly longer than those in the mild CAP group (*P* < 0.05). Moreover, respiration rate, pulmonary infiltrates, tracheal secretions, and clinical pulmonary infection scores (CPIS) in the severe group were indicative of more serious symptoms (*P* < 0.05). Among the commonly used laboratory indicators of disease severity, only white blood cell count and neutrophil counts were higher in the severe group (*P* < 0.05). Nevertheless, no significant difference was found among the other parameters (*P* > 0.05).

**Table 3:** The clinical and laboratory characteristics of the mild and severe group of children with CAP.

	Mild group (n = 97)	Severe group (n = 98)	P-value
**Ages (months)**	39.12 ± 29.36	39.34 ± 32.12	0.961
**Sex, male, n (%)**	56 (57.73)	65 (66.32)	0.216
**Weight (kg)**	14.5 (10.5, 17)	13 (10, 17)	0.166
**Height (cm)**	95.58 ± 22.01	93.92 ± 22.95	0.608
**Paroxysm duration** **before admission (days)**	5 (3, 7)	6.5 (4, 10)	0.031
**Fever duration** **before admission (days)**	3 (1, 5)	4 (1, 7)	0.019
**Cough duration** **before admission (days)**	5 (3, 8)	5 (3, 10)	0.234
**Hospital stays (days)**	4.76 ± 1.89	6.06 ± 2.53	< 0.001
**Pulse (beats/min)**	112.46 ± 15.48	112.49 ± 15.40	0.991
**Respiration rate (times/min)**	28.19 ± 6.07	31.58 ± 9.01	0.002
**Peak temperature (°C)**	38.72 ± 1.21	38.90 ± 1.18	0.310
**Pulmonary radiograph finding (%)**			< 0.001
** No infiltrate**	36 (37.11)	5 (5.10)	
** Diffuse or patch infiltrate**	56 (57.73)	37 (37.76)	
** Localized infiltrate**	5 (5.16)	56 (57.14)	
**Tracheal secretions (%)**			< 0.001
** Non or less**	31 (31.96)	25 (25.51)	
** A lot, not purulent**	61 (62.89)	35 (35.71)	
** A lot, purulent**	5 (5.15)	38 (38.78)	
**CPIS (scores)**	3.57 ± 1.55	6.10 ± 1.99	< 0.005
**hs-CRP (mg/L)**	7.62 (2.1, 20.6)	12.15 (1.43, 30)	0.148
**PCT (ng/mL)**	0.12 (0.06, 0.31)	0.12 (0.07, 0.28)	0.249
**WBC (10^9^/L)**	8.30 (6.91, 11.32)	9.87 (7.49, 13.12)	0.035
**LYM (10^9^/L)**	3.35 (2.02, 4.36)	3.03 (1.89, 4.63)	0.317
**NEU (10^9^/L)**	4.06 (2.68, 6.18)	5.13 (2.84, 8.55)	0.009
**MONO (10^9^/L)**	0.64 (0.45, 0.88)	0.75 (0.45, 0.97)	0.226
**EOS (10^9^/L)**	0.10 (0.03, 0.24)	0.10 (0.02, 0.25)	0.368
**BAS (10^9^/L)**	0.02 (0.01, 0.03)	0.02 (0.01, 0.03)	0.248

CPIS, Clinical pulmonary infection score; WBC, White blood cell count; LYM, Lymphocyte; NEU, Neutrophil; MONO, Monocytes; EOS, Eosinophil; BASO, Basophil; CXR, Chest radiography; Data comparison used Student’s test, Mann-Whitney U-test, χ^2^ Chi-squared test. P < 0.05 was considered statistically significant.

#### 3.3.2 Correlations of S100A8/A9 with laboratory indicators of severity, CXR, clinical signs, and CPIS scores among children with CAP

Since imaging is the most accurate method to determine the severity of a lung infection, we quantified the imaging findings as follows: (0: increased texture in both lungs; 1: coronal or patchy infiltrative shadows; and 2: large infiltrative or consolidated lung shadows) and assessed the correlation between S100A8/A9 levels and the degree of radiographic shadows. As shown in **Figure 2D**, the serum S100A8/A9 levels were progressively higher with increasing CXR scores. Univariate regression analysis confirmed the positive correlation between serum S100A8/A9 and CXR scores (**Table 4**). Disease onset before admission, cough duration before admission, hs-CRP, and oxygenation index were adjusted for control confounding factors. Multivariate logistic regression analysis found that serum S100A8/A9 was positively associated with CXR scores in patients with CAP (**Table 4**). The relationship between serum S100A8/A9 levels and laboratory indicators of severity, including PCT, hs-CRP, and leukocyte counts in patients with CAP, was analyzed using Spearman rank correlation. We found no significant correlation between S100A8/A9 levels and these indicators. Furthermore, we investigated clinical signs reflecting CAP severity, such as respiration rate and pulse. As shown in **Figure 2E**, serum S100A8/A9 levels positively correlated with the respiration rate (r = 0.0377, *P* < 0.01). Respiratory and pulse rates in children frequently reflect oxygenation and circulatory status. We analyzed the relationship of S100A8/A9 levels with the respiratory and pulse rates; the results revealed a weak positive correlation between S100A8/A9 levels and respiratory rate (r = 0.038, *P* < 0.01) but no correlation with pulse rate (**Figures 2F, G**). Moreover, the correlations between S100A9 levels and CPIS were explored. The CPIS represents the severity of the lung infection. We also observed a positive correlation between serum S100A9 levels and CPIS scores (r = 0.303, *P* < 0.001) (**Figure 2H**). Additionally, confounding factors were controlled and adjusted for, and multivariate logistic regression was performed. The results indicated that serum S100A9 levels positively correlated with CPIS scores (**Table 4**).

**Table 4:** Association among S100A8/A9 with clinical symptoms and prognosis in children with CAP.

	Univariate (OR/β, 95% CI)	P-value	Multivariate (OR/β, 95% CI) *	P-value
CXR scores				
**0**	1.000	/	1.000	/
**1**	2.583 (1.327, 5.029)	0.005	2.663 (1.318, 5.379)	0.006
** 2**	7.447 (2.872, 19.311)	< 0.001	7.497 (2.590, 21.700)	< 0.001
**CPIS scores**	0.944 (0.679, 1.209)	<0.001	0.252 (0.003, 0500)^a^	0.047
Hospital stays(d)				
**< 5**	1.000	/	1.000	/
** 5–9**	1.477 (1.145, 1.904)	0.003	1.486 (1.155, 1.913)	0.002
** > 9**	1.742 (1.168, 2.598)	0.006	1.599 (0.999, 2.560)	0.050

*Adjusted for hs-CRP, Peak temperature, and Oxygenation index. ^a^Adjusted for hs-CRP, Peak temperature and Chest radiography. The association of serum S100A8/A9 and clinical symptoms, hospital stays were analyzed via logistical regression analysis.

### 3.4 The predictive capacity of serum S100A8/A9 levels for disease severity in children with CAP

**Figure 3:**
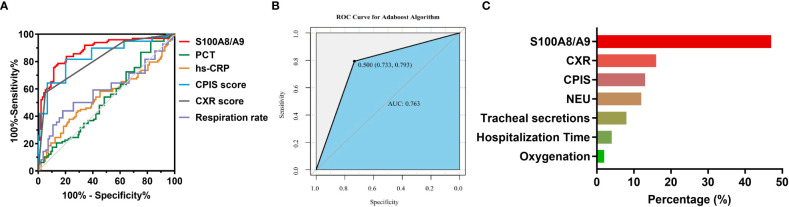
The diagnostic efficacy of S100A8/A9 in the severity of children with CAP. **(A)** ROC curves for severity prediction by S100A8/A9, PCT, hs-CRP, CPIS scores, CXR scores and respiration rate. **(B)** ROC curves for boosting tree modeling after screening for indicators affecting the severity of CAP in children. **(C)** Importance ranking of variables in a random forest model for discriminating the severity of CAP in children. NEU, Neutrophil count, Oxygenation, oxygenation index.

Using the healthy group as a control, as shown in **Supplementary Figure 1A**, the diagnostic efficacy of S100A8/A9 (AUC: 0.925, sensitivity: 0.944, and specificity: 0.730) was higher than PCT (AUC: 0.894, sensitivity: 0.815, specificity: 0.921) and hs-CRP (AUC: 0.864, sensitivity: 0.687, specificity: 0.968) in children with CAP. In addition, to investigate the differential diagnostic ability of S100A8/A9, we enrolled children with non-infectious pneumonia as control. We found that the diagnostic efficacy of S100A8/A9 (AUC: 0.685) was lower than that of hs-CRP (AUC: 0.717) but higher than that of PCT (AUC: 0.586) (**Supplementary Figure 1B**). Since the S100A8/A9 levels differed significantly between children with mild and severe CAP, we further explored the ability of S100A8/A9 and common indicators to identify an optimal marker for determining the disease severity. The results are presented in **Table 5** and **Figure 3A**: S100A8/A9 exhibited the highest diagnostic efficacy, above those of the CPIS score and CXR. The model was constructed by data cleaning and variable screening to explore the combined diagnostic efficacy to select suitable markers for condition determination. The results revealed that a classification tree model comprising S100A8/A9 and CXR had higher diagnostic accuracy (AUC: 0.875) than the independent diagnostic efficiency of S100A8/A9 (AUC: 0.862, *P* = 0.387). The diagnostic performance remained excellent, with an accuracy of 0.730 after correcting the classification tree model using a boosting algorithm (**Figure 3B**). Meanwhile, the results from a constructed random forest model revealed S100A8/A9’s superiority regarding accuracy and consistency in defining the severity of CAP in children (**Figure 3C**). Overall, these results indicate that S1000A8/A9 is significant in determining the severity of CAP in children.

**Table 5:** Evaluation of indicators reflecting the effectiveness of the diagnosis between mild CAP and severe CAP.

	AUC	Sensitivity	Specificity	Cut-off point	Youden index	*P*-value
**S100A8/A9**	0.862	0.753	0.871	1.246	0.623	< 0.001
**hs-CRP**	0.546	0.361	0.777	21.30	0.137	0.301
**PCT**	0.545	0.899	0.212	0.05	0.111	0.299
**CPIS scores**	0.835	0.643	0.887	5	0.530	< 0.001
**CXR scores**	0.815	0.871	0.948	1	0.520	< 0.001
**Respiration rate(times/min)**	0.588	0.388	0.845	32	0.233	0.034

AUC, receiver operating characteristic curve. P < 0.05 was considered statistically significant.

### 3.5 Association between the serum S100A8/A9 levels and prognosis in children with CAP

**Figure 4:**
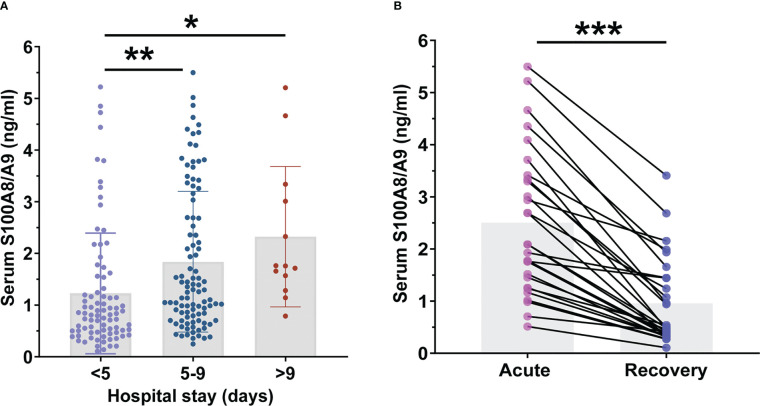
The levels of serum S100A8/A9 in community-acquired pneumonia (CAP) children with different prognosis. **(A)** The levels of serum S100A8/A9 in CAP children with different hospital stays. **(B)** Serum S100A8/A9 in children with CAP at different phase. All data were expressed as mean ± SEM. *P < 0.05, **P < 0.01, ***P < 0.001.

Serum S100A8/A9 was compared among patients with CAP with different lengths of hospital stay. As shown in **Figure 4A**, serum S100A8/A9 levels were lower in patients who stayed for < 5 days than those in patients who stayed 5–9 days and > 9 days. Furthermore, the association between serum S100A8/A9 levels and the prognosis was explored using logistic regression analysis among children with CAP. The univariate logistic regression analysis indicated that serum S100A8/A9 levels on admission positively correlated with the length of hospital stay (**Table 4**). Furthermore, multivariate logistical regression found that serum S100A8/A9 elevation prolonged the hospital stay from 5 to 9 days by 1.486 times (**Table 4**). Moreover, serum samples were collected from 28 follow-up patients who were evaluated 1 week after discharge. The serum S100A8/A9 levels were further compared among patients with CAP in the acute and recovery phase within 1 week of discharge after hospitalization. As shown in **Figure 4B**, the serum S100A8/A9 levels decreased in patients with CAP in the recovery phase compared with those in the acute phase.

## 4 Discussion

Currently, there is a lack of validated grading criteria for the severity of CAP in children (Florin et al., 2020). In this study, we found that: (1) serum S100A8/A9 level was elevated in children with pneumonia, either CAP or non-infection pneumonia (pneumonitis); (2) serum S100A8/A9 expression was higher in patients with mixed infections than in those with a single type of infection; (3) serum S100A8/A9 levels at admission were positively correlated with the severity of CAP, and were diagnostically superior for determining the severity of CAP in children compared with clinically available inflammatory markers; (4) elevation of serum S100A8/A9 on admission correlated with prolonged length of hospital stay among children with CAP. These results prove that serum S100A8/A9 is positively associated with the severity and length of hospital stay in children with CAP. Meanwhile, these findings suggest that S100A8/A9 can complement the existing severity assessment indicators for CAP in children.

Since the immune system in children is forming, susceptibility to pathogens, clinical manifestations, and disease progression in pediatrics vary from those in adults. Therefore, the reference ranges and diagnostic tipping points for severity indicators in adults with CAP do not apply to children (Kaufmann et al., 2018). In addition, ultrasound technology, more advanced imaging techniques, laboratory markers, and quantitative uniform assessment indicators should be established to facilitate the evaluation of the severity of CAP in children and make it more accurate. As the currently used clinical indicators to assess the severity of infection, hs-CRP and PCT show good efficacy in diagnosing disease severity in adults. Recent research has also shown that PCT and hs-CRP play a limited role in predicting the severity of CAP in children (Principi and Esposito, 2017). In our study, no correlation was found between PCT with CPIS scores (**Supplementary Figure 2**). PCT is more suitable for identifying children with combined pleural effusion and sepsis and guiding antibiotic dosing for pneumonia caused by a bacterial infection (Sartori et al., 2021). The efficacy of PCT was also higher in differentiating bacterial infectious pneumonia in children in our study (**Supplementary Figure 1C**). Concurrently, S100A8/A9 showed no significant difference in distinguishing the pathogenic types of pneumonia infection, which may indicate that S100A8/A9 is a non-specific indicator of inflammation. We investigated the temporal phase of secretion of PCT and S100A8/A9 (**Supplementary Figure 1D**); the results showed that the elevation peak of S100A8/A9 appeared later than that of PCT. We can determine the severity of the inflammation in the body based on the serum S100A8/A9 level measured at admission and the symptoms during paroxysm before admission. Therefore, since PCT cannot identify mild cases, combining with S100A8/A9 during the disease course could improve the diagnostic efficiency of bacterial pneumonia and predict the disease in time to guide appropriate treatment.

As an inflammatory cytokine, S100A8/A9 has been used as a valid marker for predicting the severity of various inflammatory diseases, such as inflammatory bowel disease and rheumatoid arthritis. Some have been applied to real-world clinical settings (Chimenti et al., 2013; Falvey et al., 2015). In this study, serum S100A8/A9 levels in children infected with CAP (1.59 ± 1.32 ng/mL) were five times higher than those in healthy children (0.31 ± 0.21 ng/mL) and approximately two times higher than those in children with pneumonitis (0.79 ± 0.40 ng/mL). CAP occurs mainly as an acute pulmonary infectious inflammation caused by the activation of innate immunity. The immune system directs phagocytes and killer cells to the center of inflammation by recognizing pathogen-associated molecular pattern (PAMP) molecules on pathogens and DAMP released by the immune cells. When CAP occurs due to infections, phagocytes are stimulated by PAMP molecules to secrete S100A8/A9 and migrate toward the center of inflammation (Chakraborty et al., 2017; Hudson and Lippman, 2018). Furthermore, S100A8/A9 triggers an inflammatory cascade response that exacerbates the disease. This interaction makes the level of S100A8/A9 positively correlate with the disease severity. However, in non-infectious pneumonia, the body’s immune system produces less S100A8/A9 than in CAP due to the lack of stimulation by PAMP molecules. This result may provide a basis for diagnosing children with CAP and a differential diagnosis between CAP and non-infectious pneumonia (pneumonitis).

S100A8/A9, although mainly derived from immunocytes, including neutrophils and macrophages, is also expressed as alveolar cell type 2, vascular endothelial cells during trauma, infection, heat, and many other inflammatory processes (Wang et al., 2018). In cases of severe localized infection, S100A8/A9 is secreted extracellularly to recruit large numbers of immune cells toward the center of infection. Simultaneously, the systemic inflammatory cascade is triggered by the interaction of over-secreted cytokines and S100A8/A9, disrupting the body’s immune homeostasis and creating a severe “inflammatory storm.” Our previous study also revealed the elevated expression of S100A8/A9 in the alveolar lavage fluid of mice infected with Streptococcus pneumoniae, which was consistent with previous reports (Achouiti et al., 2014). This confirms the involvement of S100A8/A9 in the pathophysiological process of CAP. However, the mechanism of S100A8/A9 secretion and action in CAP is unclear. It has been demonstrated that S100A8/A9 secretion by lipopolysaccharide activates the caspase-4/5 inflammasome (Coveney et al., 2015). Our study revealed that serum S100A8/A9 was not differentially expressed in children infected with different single types of pathogens. However, there is a significant difference in mixed infections with multiple types of pathogens compared with single types of infection. This suggests that the release of S100A8/A9 may not only result from the activation of pattern recognition receptors; however, interactions between microbially expressed virulence factors may facilitate cellular secretion of S100A8/A9 (Grousd et al., 2019). Therefore, we will investigate the role and secretion mechanism of S100A8/A9 in CAP to provide theoretical support for identifying severe disease and targeted host-directed therapy.

This study had some limitations. First, the sample size was relatively small, and all participants were from a single center. Second, since most of the non-infectious pneumonia samples we received were derived from acute bronchial foreign bodies, a differential bias might have been caused by the shorter duration of the disease. Third, our study did not compare the difference in diagnostic efficacy with other indicators, such as IL-6, SAA, FER, and HBP, that also indicate the severity of the infection. Finally, the systemic S100A8/A9 level was measured in the serum, and the local S100A8/A9 levels, such as those in the sputum, lungs, and bronchoalveolar lavage fluid, were not tested to verify our findings. Therefore, we will further conduct a multi-center, multi-indicator evaluation of S100A8/A9 for diagnosing the severity of CAP and explore convenient and improved methods other than the colloidal gold method currently used clinically so that S100A8/A9 can be better used for assessing the severity of CAP.

## 5 Conclusion

Serum S100A8/A9 levels may aid in determining the diagnosis and severity of CAP in children, and S100A8/A9 levels of ≥ 1.246 ng/mL may be a more valuable cut-off value for severe CAP, supporting it as a novel and intriguing biomarker for pneumonia to the monitor children patients’ response and predict the condition, which may have implications for more accurate medical triage decisions.

## Data availability statement

The original contributions presented in the study are included in the article/**Supplementary Materials**, further inquiries can be directed to the corresponding author/s.

## Ethics statement

The studies involving human participants were reviewed and approved by the Medical Ethical Committee of Wuhan Children’s Hospital, Huazhong University of Science and Technology (2022R048-E01). Written informed consent to participate in this study was provided by the participants’ legal guardian/next of kin.

## Author contributions

SX, HMZ, CHY and YX: study conception and design. JW, WBT, SX, FH and CQZ: data acquisition. SHZ and FH: investigation, assessment of clinical significance of the method. CY and SX: analysis and data interpretation. JW and SX: drafting of the manuscript. All authors contributed to the article and approved the submitted version.
