# Supplementary material for: Serum level of S100A8/A9 as a biomarker for establishing the diagnosis and severity of community-acquired pneumonia in children
Source: Front Cell Infect Microbiol. 2023 Apr 27;13:1139556. doi: 10.3389/fcimb.2023.1139556 (PMC10172663; doi:10.3389/fcimb.2023.1139556)
Supplement: Supplementary file 1 [file DataSheet_1.docx]

Supplementary Material

Serum level of S100A8/A9 as a biomarker for establishing the diagnosis and severity of community-acquired pneumonia in children

**Si Xie^1^*, Jun Wang, Wenbin Tuo**

**Correspondence:** Hongmin Zhu：zhuhongmin@zgwhfe.com
Chunhui Yuan：chunhuii.yuen@whu.edu.cn

Yun Xiang ：xiangyun5272008@163.com

# Supplementary Figures

## Supplementary Figure 1


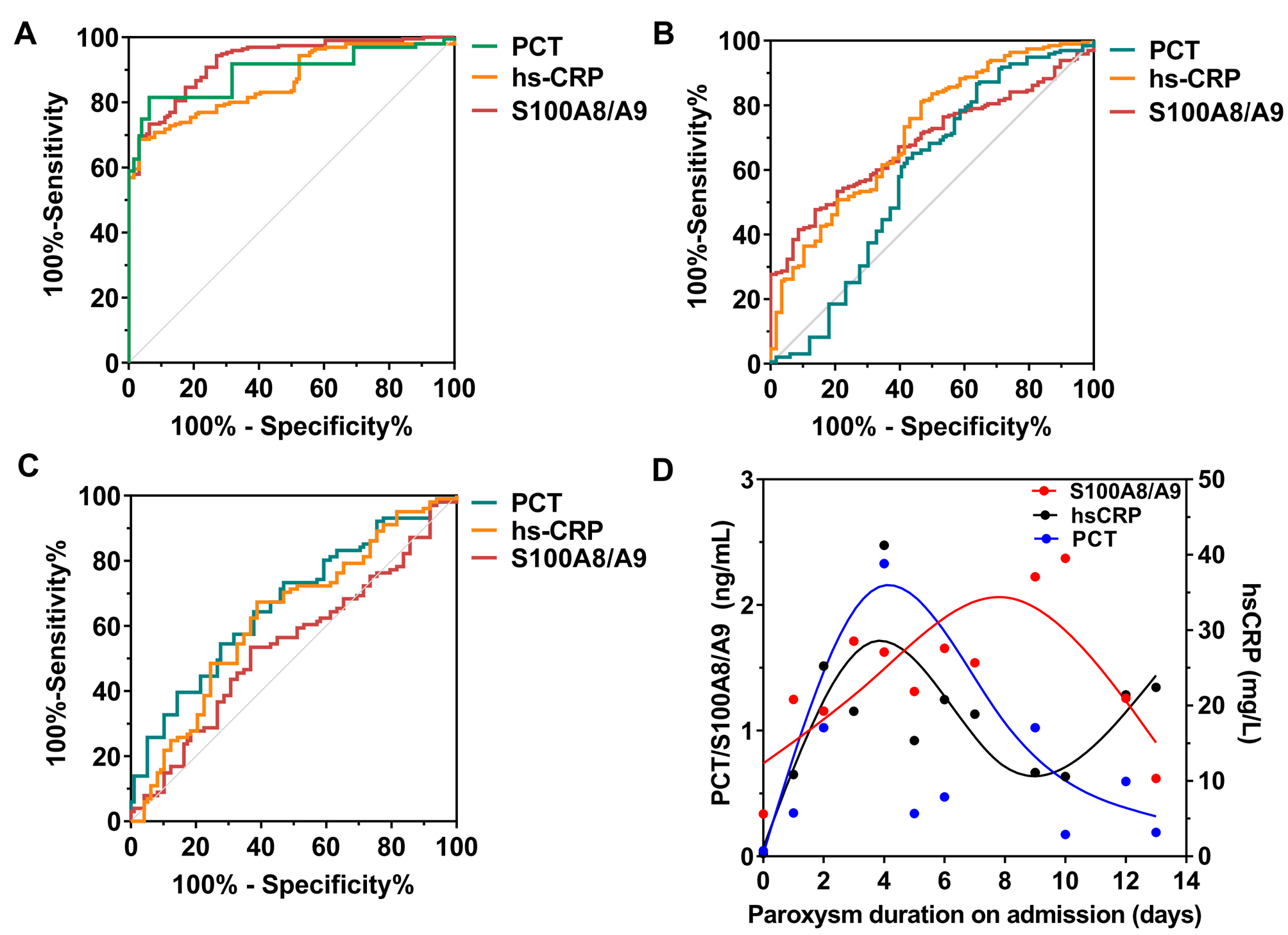


**Supplementary Figure 1.** **Diagnostic efficacy of S100A8/A9, procalcitonin (PCT), hypersensitive C-reactive protein (hs-CRP) and changes in secretion time phases.** (**A**) Diagnostic efficacy of S100A8/A9, PCT, hs-CRP in diagnosing children with CAP. (**B**) Diagnostic efficacy of S100A8/A9, PCT, hs-CRP in differential diagnosis of children with CAP. (**C**) Diagnostic efficacy of S100A8/A9, PCT, hs-CRP in differentiating bacterial CAP from other types of CAP. (**D**) Secretion time phases of S100A8/A9, PCT, hs-CRP.

## Supplementary Figure 2


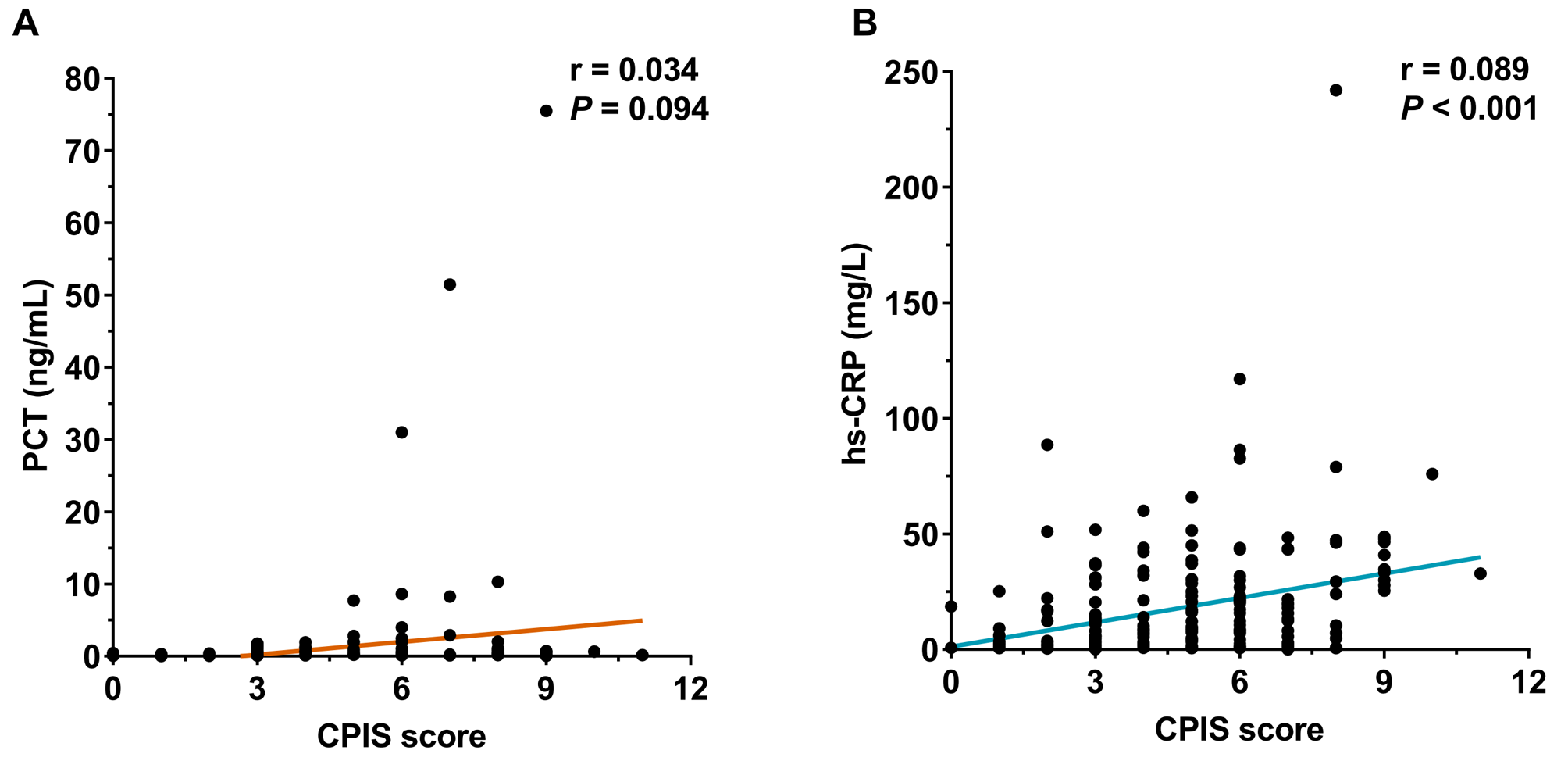


**Supplementary Figure 2. Correlation of PCT and hs-CRP with the severity scores (CPIS score) of pulmonary infections (A ─ B).**
